# VARIATION AND CONSISTENCY IN ALLOCATION OF HCBS: MASSACHUSETTS AGING SERVICES ACCESS POINTS

**DOI:** 10.1093/geroni/igac059.2156

**Published:** 2022-12-20

**Authors:** Signe Flieger, Gabrielle Katz, Christine Bishop

**Affiliations:** Tufts University School of Medicine, Boston, Massachusetts, United States; Brandeis University, Waltham, Massachusetts, United States; Brandeis University, Waltham, Massachusetts, United States

## Abstract

Massachusetts’ 27 Aging Services Access Points (ASAPs) are nonprofit organizations responsible for guiding clients and families in their geographic service areas through a maze of financial and functional eligibility criteria to receive needed home and community-based services (HCBS). These services aim to sustain function and promote engagement for older adults and persons with disabilities, and to maintain individuals in the community rather than in residential settings. Payment for HCBS flows from numerous state and Federal funding programs, including but not limited to state Medicaid waivers and Older American Act programs. We conducted semi-structured interviews with 10 ASAP directors and gathered information from documentary sources to delineate the pathways to funded HCBS that these agencies open for clients. We identified variation in the 27 ASAPs’ contractual relationships with service providers. State data revealed variation in budgetary resources and area population characteristics. These nonprofit corporations appear to flexibly adapt to local conditions and needs. Understanding variation across ASAPs will support analysis of client data to examine whether ASAP processes result in different service outcomes for similar clients. Alternate payment models for health services (managed care, managed long-term services and supports, accountable care organizations) are broadening their reach, encouraging health systems to recognize the importance of functional supports and services. Some health systems are charging their own dedicated units with assigning HCBS to enrollees, avoiding the ASAPs. Understanding and modeling the allocation of services by the ASAPs will allow investigation of trends in services for persons with varying needs under various payment methods.

